# 6,7-Di­chloro-2,3-bis(pyridin-2-yl)quinox­aline

**DOI:** 10.1107/S2056989015000055

**Published:** 2015-01-10

**Authors:** Guy Crundwell, Neil M. Glagovich, Melissa E. King

**Affiliations:** a Department of Chemistry & Biochemistry, Central Connecticut State University, New Britain, CT 06053, USA

**Keywords:** crystal structure, quinoxaline

## Abstract

The title compound, C_18_H_10_Cl_2_N_4_, synthesized by the condensation reaction between 4,5-di­chloro­benzene-1,2-di­amine and 1,2-di(pyridin-2-yl)ethane-1,2-dione in boiling acetic acid, has a nearly planar quinoxaline moiety [maximum deviation = 0.070 (1) Å] whose mean plane makes dihedral angles of 40.51 (2) and 39.29 (3)° with the pyridine rings. Within the unit cell, there are no classical hydrogen bonds. Molecules in the structure pack with π–π stacking contacts between the quinoxaline units and nearby pyridine rings with an intercentroid distance of 3.7676 (9) Å.

## Related literature   

For the synthesis of the title compound, see: Imeri *et al.* (2013 ▸). For the structures of similar compounds, see: Woźniak (1991 ▸); Rasmussen *et al.* (1990 ▸); Crundwell *et al.* (2010 ▸, 2014 ▸); Jaso *et al.* (2005 ▸); Bu *et al.* (2001 ▸); Cantalupo *et al.* (2010 ▸); Crundwell (2013 ▸).
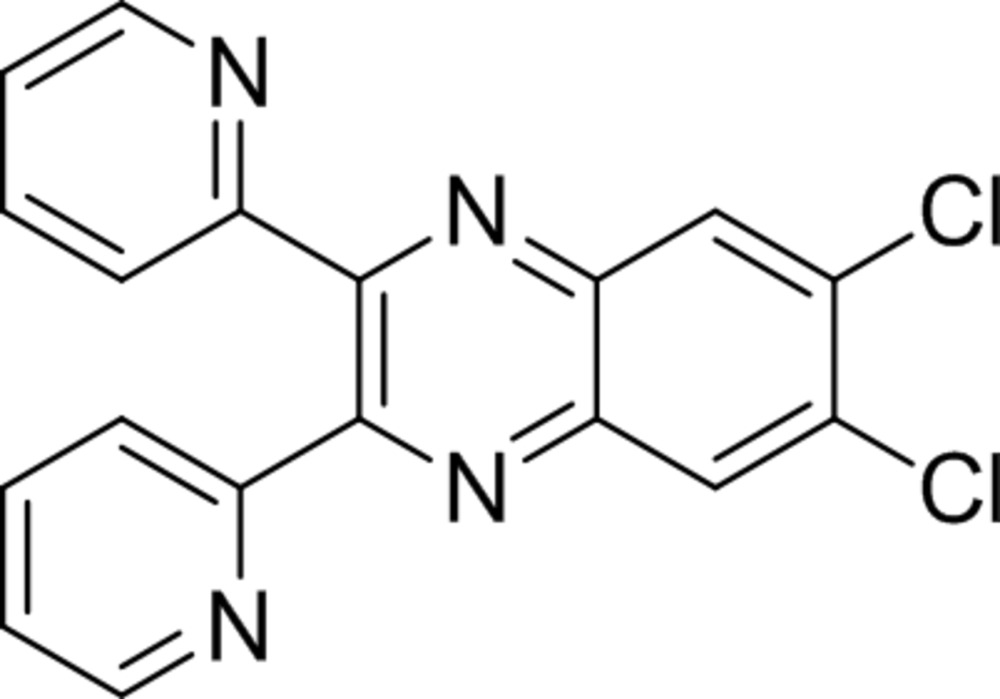


## Experimental   

### Crystal data  

C_18_H_10_Cl_2_N_4_
*M* *_r_* = 353.20
Orthorhombic, 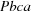

*a* = 7.1921 (5) Å
*b* = 18.072 (3) Å
*c* = 24.093 (4) Å
*V* = 3131.6 (8) Å^3^
*Z* = 8
Mo *K*α radiation
μ = 0.42 mm^−1^
*T* = 110 K
0.23 × 0.12 × 0.09 mm

### Data collection  

Oxford Diffraction Xcalibur, Sapphire3 diffractometer
Absorption correction: multi-scan (*CrysAlis RED*; Oxford Diffraction, 2009 ▸) *T*_min_ = 0.971, *T*_max_ = 1.000
24047 measured reflections
6227 independent reflections
3818 reflections with *I* > 2σ(*I*)
*R*_int_ = 0.031

### Refinement  

*R*[*F* ^2^ > 2σ(*F* ^2^)] = 0.034
*wR*(*F* ^2^) = 0.086
*S* = 0.90
6227 reflections
217 parameters
H-atom parameters constrained
Δρ_max_ = 0.45 e Å^−3^
Δρ_min_ = −0.24 e Å^−3^

### 

Data collection: *CrysAlis CCD* (Oxford Diffraction, 2009 ▸); cell refinement: *CrysAlis RED* (Oxford Diffraction, 2009 ▸); data reduction: *CrysAlis RED*; program(s) used to solve structure: *SHELXS97* (Sheldrick, 2008 ▸); program(s) used to refine structure: *SHELXL97* (Sheldrick, 2008 ▸); molecular graphics: *PLATON* (Spek, 2009 ▸); software used to prepare material for publication: *SHELXTL* (Sheldrick, 2008 ▸).

## Supplementary Material

Crystal structure: contains datablock(s) I, New_Global_Publ_Block. DOI: 10.1107/S2056989015000055/hg5423sup1.cif

Structure factors: contains datablock(s) I. DOI: 10.1107/S2056989015000055/hg5423Isup2.hkl

Supporting information file. DOI: 10.1107/S2056989015000055/hg5423Isup3.cml

**Fig. 1.**
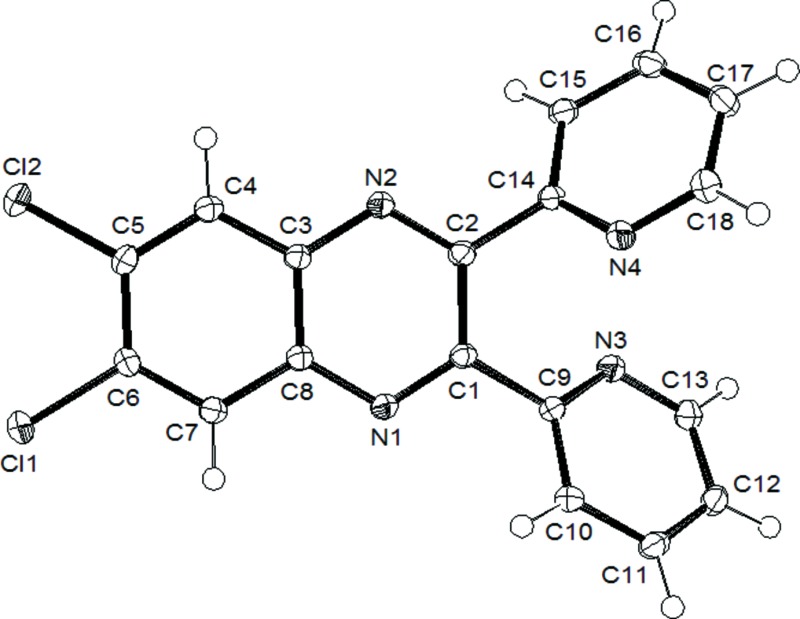
A view of the title compound (Spek, 2009). Displacement ellipsoids are drawn at the 50% probability level.

. DOI: 10.1107/S2056989015000055/hg5423fig1.tif
A view of the title compound (Spek, 2009). Displacement ellipsoids are drawn at the 50% probability level.

CCDC reference: 1041827

Additional supporting information:  crystallographic information; 3D view; checkCIF report

## supplementary crystallographic information

### S1. Comment

Quinoxalines, like the title compound C_18_H_10_Cl_2_N_4_, have interesting characteristics including antimicrobial activity (Jaso *et al.*, 2005). Our interest in quinoxalines results from their simple synthesis, and the proclivity with which X-ray quality crystals can be grown (Imeri *et al.*, 2013; Crundwell *et al.*, 2010; Cantalupo *et al.*, 2010; Wozniak, 1991). We have also found that quinoxalines make interesting ligands when combined with metals, especially silver(I) salts (Rasmussen *et al.*, 1990; Bu *et al.*, 2001; Crundwell, 2013; Crundwell *et al.*, 2014).

The title compound, C_18_H_10_Cl_2_N_4_, has a nearly planar quinoxaline moiety (Fig. 1). The pyridine rings make angles of 40.52 (2)° and 39.32 (3)° with respect to the mean plane of the quinoxaline. All bond lengths and angles lie within expected values. Within the unit cell, there are no classical hydrogen bonds. The molecules pack in offset layers with closest contacts between quinoxalines and nearby pyridine rings.

### S2. Experimental

The title compound was synthesized in a manner similar to related compounds Imeri *et al.* (2013). To a 50 ml round bottom flask equipped with a reflux condenser was combined 0.2685 g (1.265 mmol) 1,2-di(pyridin-2-yl)ethane-1,2-dione, 0.3445 g (1.946 mmol) 4,5-dichlorobenzene-1,2-diamine and 20 ml glacial acetic acid. The resulting mixture was heated to reflux for 24 h. After this time, the resulting solution was poured over ice. The resulting beige solid was filtered and recrystallized from methanol, which produced 0.299 g of the title compound as a shiny, beige solid (67%). *R*_f_ 0.36 (SiO_2_, ethyl acetate); m.p. 468 K; IR (ATR-FTIR) 3077, 3046, 3008, 1585, 1474, 1391, 1341, 1278, 1110, 1078, 1002, 965, 860, 790, 743, 599, 547 cm^-1^; ^1^H NMR (300 MHz, CDCl_3_) δ 8.38 (s, 2H), 8.38 (dt, 2H J = 5, 1 Hz), 8.00 (dt, 2H, J = 8, 1 Hz), 7.87 (td, 2H, J = 8, 1 Hz), 7.29 (td, 2H, J = 5, 1 Hz); ^13^C NMR (300 MHz, CDCl_3_) δ 156.87, 153.53, 148.58, 139.84, 136.74, 135.10, 129.97, 124.17, 123.29; UV/Vis (CH_2_Cl_2_; λ_max_ (logε)) 348 nm (12668), 275 nm (24188) 253 nm (43604); MS calculated for C_18_H_10_Cl_2_N_4_: *M*^+^: 352, measured: 352.

### S3. Refinement

Carbon-bound H-atoms were placed in calculated positions (C—H = 0.93 Å) and were included in the refinement in the riding model approximation, with *U_iso_*(H) = 1.2*U_eq_*(C).

### Crystal data

**Table:**

C_18_H_10_Cl_2_N_4_	*D*_x_ = 1.498 Mg m^−^^3^
*M**_r_* = 353.20	Melting point: 468 K
Orthorhombic, *P**b**c**a*	Mo *K*α radiation, λ = 0.71073 Å
Hall symbol: -P 2ac 2ab	Cell parameters from 9283 reflections
*a* = 7.1921 (5) Å	θ = 3.8–34.6°
*b* = 18.072 (3) Å	µ = 0.42 mm^−^^1^
*c* = 24.093 (4) Å	*T* = 110 K
*V* = 3131.6 (8) Å^3^	Needle, brown
*Z* = 8	0.23 × 0.12 × 0.09 mm
*F*(000) = 1440	

### Data collection

**Table:**

Oxford Diffraction Xcalibur, Sapphire3 diffractometer	6227 independent reflections
Radiation source: fine-focus sealed tube	3818 reflections with *I* > 2σ(*I*)
Graphite monochromator	*R*_int_ = 0.031
Detector resolution: 16.1790 pixels mm^-1^	θ_max_ = 34.7°, θ_min_ = 4.0°
ω scans	*h* = −10→11
Absorption correction: multi-scan (*CrysAlis RED*; Oxford Diffraction, 2009)	*k* = −27→27
*T*_min_ = 0.971, *T*_max_ = 1.000	*l* = −36→12
24047 measured reflections	

### Refinement

**Table:**

Refinement on *F*^2^	Primary atom site location: structure-invariant direct methods
Least-squares matrix: full	Secondary atom site location: difference Fourier map
*R*[*F*^2^ > 2σ(*F*^2^)] = 0.034	Hydrogen site location: inferred from neighbouring sites
*wR*(*F*^2^) = 0.086	H-atom parameters constrained
*S* = 0.90	*w* = 1/[σ^2^(*F*_o_^2^) + (0.0479*P*)^2^] where *P* = (*F*_o_^2^ + 2*F*_c_^2^)/3
6227 reflections	(Δ/σ)_max_ = 0.002
217 parameters	Δρ_max_ = 0.45 e Å^−^^3^
0 restraints	Δρ_min_ = −0.24 e Å^−^^3^

### Special details

**Table:**

Experimental. Carbon-bound H-atoms were placed in calculated positions (C—H = 0.93 Å) and were included in the refinement in the riding model approximation, with *U_iso_*(H) = 1.2*U_eq_*(C).
Geometry. All esds (except the esd in the dihedral angle between two l.s. planes) are estimated using the full covariance matrix. The cell esds are taken into account individually in the estimation of esds in distances, angles and torsion angles; correlations between esds in cell parameters are only used when they are defined by crystal symmetry. An approximate (isotropic) treatment of cell esds is used for estimating esds involving l.s. planes.
Refinement. Refinement of *F*^2^ against ALL reflections. The weighted *R*-factor *wR* and goodness of fit *S* are based on *F*^2^, conventional *R*-factors *R* are based on *F*, with *F* set to zero for negative *F*^2^. The threshold expression of *F*^2^ > σ(*F*^2^) is used only for calculating *R*-factors(gt) *etc*. and is not relevant to the choice of reflections for refinement. *R*-factors based on *F*^2^ are statistically about twice as large as those based on *F*, and *R*- factors based on ALL data will be even larger.

### Fractional atomic coordinates and isotropic or equivalent isotropic displacement parameters (Å^2^)

**Table:**

	*x*	*y*	*z*	*U*_iso_*/*U*_eq_	
Cl2	0.32732 (4)	0.426900 (14)	1.127570 (12)	0.02132 (7)	
Cl1	0.47814 (4)	0.573896 (15)	1.184200 (12)	0.02235 (7)	
N1	0.40209 (13)	0.70156 (5)	0.99845 (4)	0.01556 (18)	
C6	0.41314 (16)	0.57361 (6)	1.11493 (5)	0.0160 (2)	
N2	0.22634 (13)	0.57626 (5)	0.95171 (4)	0.01546 (18)	
C14	0.15721 (15)	0.64197 (6)	0.86826 (5)	0.0142 (2)	
N4	0.06670 (13)	0.70481 (5)	0.85634 (4)	0.0180 (2)	
N3	0.45961 (14)	0.75394 (5)	0.86134 (4)	0.0178 (2)	
C3	0.29491 (15)	0.57490 (5)	1.00477 (5)	0.0144 (2)	
C5	0.34195 (15)	0.50820 (6)	1.09007 (5)	0.0160 (2)	
C15	0.16747 (16)	0.58249 (6)	0.83174 (5)	0.0174 (2)	
H15	0.2258	0.5387	0.8422	0.021*	
C1	0.34541 (15)	0.70080 (5)	0.94668 (5)	0.0143 (2)	
C2	0.24614 (15)	0.63859 (5)	0.92372 (5)	0.0144 (2)	
C7	0.42943 (15)	0.63766 (6)	1.08503 (5)	0.0162 (2)	
H7	0.4772	0.6801	1.1015	0.019*	
C9	0.39540 (15)	0.76674 (5)	0.91270 (5)	0.0146 (2)	
C4	0.28220 (15)	0.50930 (6)	1.03626 (5)	0.0166 (2)	
H4	0.2330	0.4667	1.0204	0.020*	
C18	−0.01188 (17)	0.70972 (6)	0.80610 (5)	0.0210 (2)	
H18	−0.0776	0.7526	0.7976	0.025*	
C8	0.37327 (15)	0.63874 (6)	1.02907 (5)	0.0149 (2)	
C11	0.41978 (16)	0.89793 (6)	0.90239 (5)	0.0188 (2)	
H11	0.4055	0.9458	0.9159	0.023*	
C10	0.37737 (16)	0.83738 (5)	0.93523 (5)	0.0169 (2)	
H10	0.3376	0.8437	0.9716	0.020*	
C16	0.08919 (17)	0.58966 (6)	0.77953 (5)	0.0211 (2)	
H16	0.0971	0.5513	0.7539	0.025*	
C13	0.50436 (16)	0.81321 (6)	0.83081 (5)	0.0198 (2)	
H13	0.5518	0.8054	0.7954	0.024*	
C12	0.48406 (16)	0.88553 (6)	0.84893 (5)	0.0196 (2)	
H12	0.5130	0.9250	0.8257	0.024*	
C17	−0.00125 (16)	0.65502 (6)	0.76609 (5)	0.0222 (2)	
H17	−0.0533	0.6619	0.7311	0.027*	

### Atomic displacement parameters (Å^2^)

**Table:**

	*U*^11^	*U*^22^	*U*^33^	*U*^12^	*U*^13^	*U*^23^
Cl2	0.02666 (15)	0.01669 (12)	0.02059 (14)	−0.00209 (10)	−0.00040 (11)	0.00626 (11)
Cl1	0.03104 (16)	0.02148 (13)	0.01452 (13)	−0.00167 (11)	−0.00438 (11)	0.00270 (11)
N1	0.0188 (5)	0.0130 (4)	0.0149 (5)	−0.0012 (3)	0.0003 (4)	−0.0006 (3)
C6	0.0167 (5)	0.0180 (5)	0.0132 (5)	0.0002 (4)	−0.0012 (4)	0.0006 (4)
N2	0.0191 (4)	0.0133 (4)	0.0140 (4)	−0.0002 (3)	−0.0001 (4)	−0.0006 (3)
C14	0.0160 (5)	0.0131 (4)	0.0136 (5)	−0.0023 (4)	0.0001 (4)	−0.0005 (4)
N4	0.0206 (5)	0.0163 (4)	0.0170 (5)	0.0008 (4)	−0.0022 (4)	−0.0002 (4)
N3	0.0221 (5)	0.0158 (4)	0.0157 (5)	−0.0014 (4)	0.0004 (4)	−0.0007 (4)
C3	0.0161 (5)	0.0133 (4)	0.0138 (5)	−0.0004 (4)	0.0004 (4)	−0.0007 (4)
C5	0.0168 (5)	0.0138 (5)	0.0174 (5)	0.0016 (4)	0.0020 (4)	0.0027 (4)
C15	0.0207 (5)	0.0145 (5)	0.0169 (5)	−0.0035 (4)	0.0017 (4)	−0.0011 (4)
C1	0.0167 (5)	0.0115 (4)	0.0148 (5)	0.0007 (4)	0.0008 (4)	−0.0011 (4)
C2	0.0169 (5)	0.0132 (4)	0.0133 (5)	−0.0001 (4)	0.0018 (4)	−0.0016 (4)
C7	0.0178 (5)	0.0155 (5)	0.0154 (5)	−0.0018 (4)	−0.0002 (4)	−0.0017 (4)
C9	0.0161 (5)	0.0129 (4)	0.0148 (5)	−0.0005 (4)	−0.0019 (4)	0.0004 (4)
C4	0.0195 (5)	0.0131 (5)	0.0172 (6)	−0.0013 (4)	0.0001 (4)	−0.0014 (4)
C18	0.0218 (6)	0.0210 (5)	0.0203 (6)	0.0008 (5)	−0.0029 (5)	0.0020 (5)
C8	0.0169 (5)	0.0134 (4)	0.0143 (5)	−0.0001 (4)	0.0005 (4)	−0.0003 (4)
C11	0.0194 (6)	0.0126 (5)	0.0245 (6)	−0.0005 (4)	−0.0029 (5)	0.0000 (4)
C10	0.0190 (5)	0.0149 (5)	0.0167 (6)	−0.0006 (4)	−0.0009 (4)	−0.0022 (4)
C16	0.0263 (6)	0.0211 (5)	0.0158 (6)	−0.0070 (5)	0.0019 (5)	−0.0050 (4)
C13	0.0232 (6)	0.0202 (5)	0.0160 (6)	−0.0021 (4)	0.0005 (5)	0.0021 (4)
C12	0.0215 (6)	0.0165 (5)	0.0208 (6)	−0.0029 (4)	−0.0020 (5)	0.0050 (4)
C17	0.0235 (6)	0.0275 (6)	0.0155 (6)	−0.0074 (5)	−0.0045 (5)	0.0012 (5)

### Geometric parameters (Å, º)

**Table:**

Cl2—C5	1.7281 (11)	C1—C2	1.4420 (15)
Cl1—C6	1.7332 (12)	C1—C9	1.4897 (15)
N1—C1	1.3124 (14)	C7—C8	1.4075 (15)
N1—C8	1.3697 (14)	C7—H7	0.9300
C6—C7	1.3685 (15)	C9—C10	1.3934 (14)
C6—C5	1.4207 (15)	C4—H4	0.9300
N2—C2	1.3206 (13)	C18—C17	1.3828 (17)
N2—C3	1.3703 (15)	C18—H18	0.9300
C14—N4	1.3402 (13)	C11—C10	1.3843 (15)
C14—C15	1.3912 (15)	C11—C12	1.3869 (17)
C14—C2	1.4826 (16)	C11—H11	0.9300
N4—C18	1.3390 (15)	C10—H10	0.9300
N3—C13	1.3386 (14)	C16—C17	1.3869 (17)
N3—C9	1.3411 (15)	C16—H16	0.9300
C3—C8	1.4112 (14)	C13—C12	1.3856 (16)
C3—C4	1.4107 (15)	C13—H13	0.9300
C5—C4	1.3658 (16)	C12—H12	0.9300
C15—C16	1.3841 (17)	C17—H17	0.9300
C15—H15	0.9300		
			
C1—N1—C8	117.15 (9)	N3—C9—C1	116.88 (9)
C7—C6—C5	120.84 (11)	C10—C9—C1	119.77 (10)
C7—C6—Cl1	118.78 (8)	C5—C4—C3	120.16 (10)
C5—C6—Cl1	120.38 (8)	C5—C4—H4	119.9
C2—N2—C3	116.93 (9)	C3—C4—H4	119.9
N4—C14—C15	123.03 (10)	N4—C18—C17	124.02 (11)
N4—C14—C2	115.94 (9)	N4—C18—H18	118.0
C15—C14—C2	121.03 (10)	C17—C18—H18	118.0
C18—N4—C14	117.06 (10)	N1—C8—C3	120.97 (10)
C13—N3—C9	116.86 (9)	N1—C8—C7	118.94 (10)
N2—C3—C8	121.07 (9)	C3—C8—C7	120.05 (10)
N2—C3—C4	119.58 (9)	C10—C11—C12	118.46 (10)
C8—C3—C4	119.33 (10)	C10—C11—H11	120.8
C4—C5—C6	120.10 (10)	C12—C11—H11	120.8
C4—C5—Cl2	119.30 (8)	C11—C10—C9	118.77 (11)
C6—C5—Cl2	120.59 (9)	C11—C10—H10	120.6
C16—C15—C14	118.74 (10)	C9—C10—H10	120.6
C16—C15—H15	120.6	C15—C16—C17	118.86 (11)
C14—C15—H15	120.6	C15—C16—H16	120.6
N1—C1—C2	121.77 (9)	C17—C16—H16	120.6
N1—C1—C9	116.03 (9)	N3—C13—C12	123.81 (11)
C2—C1—C9	122.16 (10)	N3—C13—H13	118.1
N2—C2—C1	121.50 (10)	C12—C13—H13	118.1
N2—C2—C14	116.67 (9)	C13—C12—C11	118.69 (10)
C1—C2—C14	121.82 (9)	C13—C12—H12	120.7
C6—C7—C8	119.43 (10)	C11—C12—H12	120.7
C6—C7—H7	120.3	C18—C17—C16	118.17 (11)
C8—C7—H7	120.3	C18—C17—H17	120.9
N3—C9—C10	123.33 (10)	C16—C17—H17	120.9
			
C15—C14—N4—C18	−1.81 (16)	N1—C1—C9—N3	135.70 (11)
C2—C14—N4—C18	178.80 (10)	C2—C1—C9—N3	−42.28 (15)
C2—N2—C3—C8	3.50 (15)	N1—C1—C9—C10	−43.17 (15)
C2—N2—C3—C4	−178.38 (10)	C2—C1—C9—C10	138.85 (11)
C7—C6—C5—C4	−2.44 (17)	C6—C5—C4—C3	1.47 (17)
Cl1—C6—C5—C4	176.69 (9)	Cl2—C5—C4—C3	−179.29 (8)
C7—C6—C5—Cl2	178.33 (9)	N2—C3—C4—C5	−176.88 (10)
Cl1—C6—C5—Cl2	−2.54 (13)	C8—C3—C4—C5	1.27 (16)
N4—C14—C15—C16	3.48 (17)	C14—N4—C18—C17	−1.49 (17)
C2—C14—C15—C16	−177.17 (10)	C1—N1—C8—C3	3.28 (15)
C8—N1—C1—C2	3.95 (15)	C1—N1—C8—C7	−179.04 (10)
C8—N1—C1—C9	−174.03 (9)	N2—C3—C8—N1	−7.34 (16)
C3—N2—C2—C1	3.71 (15)	C4—C3—C8—N1	174.53 (10)
C3—N2—C2—C14	−174.77 (9)	N2—C3—C8—C7	175.00 (10)
N1—C1—C2—N2	−7.87 (16)	C4—C3—C8—C7	−3.13 (16)
C9—C1—C2—N2	169.99 (10)	C6—C7—C8—N1	−175.51 (10)
N1—C1—C2—C14	170.53 (10)	C6—C7—C8—C3	2.19 (17)
C9—C1—C2—C14	−11.61 (15)	C12—C11—C10—C9	−1.98 (17)
N4—C14—C2—N2	136.57 (10)	N3—C9—C10—C11	2.96 (17)
C15—C14—C2—N2	−42.83 (15)	C1—C9—C10—C11	−178.25 (10)
N4—C14—C2—C1	−41.91 (14)	C14—C15—C16—C17	−1.84 (17)
C15—C14—C2—C1	138.69 (11)	C9—N3—C13—C12	−1.45 (17)
C5—C6—C7—C8	0.57 (17)	N3—C13—C12—C11	2.30 (18)
Cl1—C6—C7—C8	−178.57 (9)	C10—C11—C12—C13	−0.46 (17)
C13—N3—C9—C10	−1.23 (17)	N4—C18—C17—C16	2.97 (18)
C13—N3—C9—C1	179.95 (10)	C15—C16—C17—C18	−1.16 (17)
