# Supplementary figures and images for: 6,7-Di­chloro-2,3-bis(pyridin-2-yl)quinox­aline
Source: Acta Crystallogr E Crystallogr Commun. 2015 Jan 10;71(Pt 2):o107. doi: 10.1107/S2056989015000055 (PMC4384551; doi:10.1107/S2056989015000055)

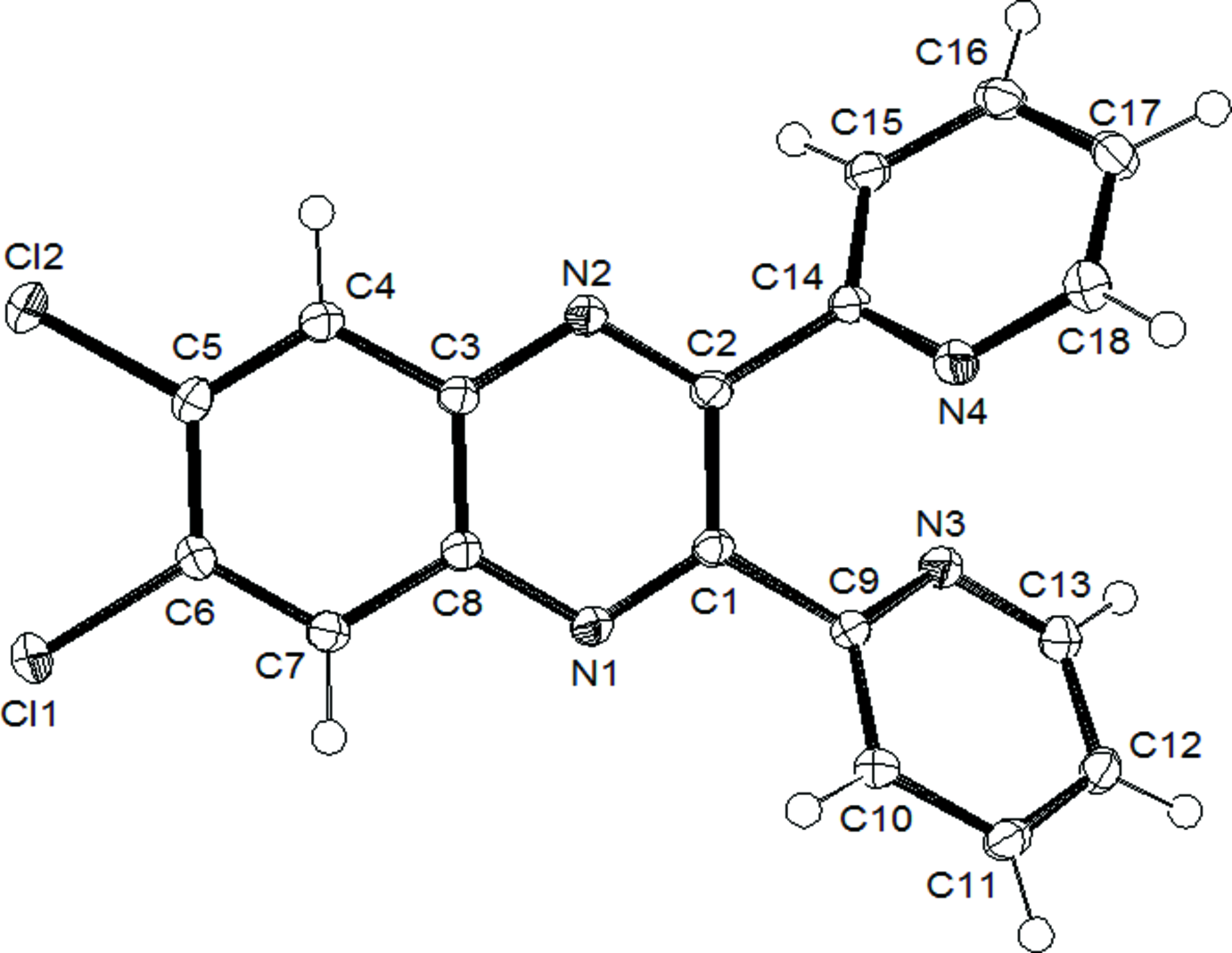

Supplement: Supplementary file 4 [file e-71-0o107-fig1.tif]
